# Laser digital patterning of conductive electrodes using metal oxide nanomaterials

**DOI:** 10.1186/s40580-020-00232-9

**Published:** 2020-07-06

**Authors:** Vu Binh Nam, Trinh Thi Giang, Sangmo Koo, Junsuk Rho, Daeho Lee

**Affiliations:** 1 Department of Chemical Engineering, Pohang University of Science and Technology (POSTECH), Pohang, 37673 South Korea; 2 Laser and Thermal Engineering Lab, Department of Mechanical Engineering, Gachon University, Seongnam, 13120 South Korea; 3 Advanced Laser Fabrication Systems Lab, Department of Mechanical Engineering, Incheon National University, Incheon, 22012 South Korea; 4 Department of Mechanical Engineering, Pohang University of Science and Technology (POSTECH), Pohang, 37673 South Korea

**Keywords:** Laser, Digital patterning, ITO, ZnO, CuO, NiO, Reductive sintering

## Abstract

As an alternative approach to the conventional deposition and photolithographic processes, the laser digital patterning (LDP) process, which is also known as the laser direct writing process, has attracted considerable attention because it is a non-photolithographic, non-vacuum, on-demand, and cost-effective electrode fabrication route that can be applied to various substrates, including heat-sensitive flexible substrates. The LDP process was initially developed using noble metal nanoparticles (NPs) such as Au and Ag because such materials are free from oxidation even in a nanosize configuration. Thus, the NPs must be fused together to form continuous conductive structures upon laser irradiation. However, common metals are easily oxidized at the nanoscale and exist in oxidized forms owing to the extremely large surface-to-volume ratio of NPs. Therefore, to fabricate conductive electrodes using common metal NPs via the LDP process, laser irradiation should be used to sinter the NPs and simultaneously induce additional photochemical reactions, such as reduction, and defect structure modification to increase the conductivity of the electrodes. This review summarizes recent studies on the LDP process in which metal oxide NPs, such as ITO, ZnO, CuO, and NiO, were exclusively utilized for fabricating conductive electrodes. The outlook of the LDP process for these materials is also discussed as a method that can be used together with or as a replacement for conventional ones to produce next-generation transparent conductors, sensors, and electronics.

## Introduction

Lasers are widely used for various purposes, including presentation aids in daily life and industrial, medical, and scientific research and development because they are effective sources of heat [1–4] and illumination [5–9]. Lasers have the following unique characteristics that differentiate them from other heat and illumination systems. First, a laser can emit collimated light of a single wavelength owing to its spatial and temporal coherence. For this reason, a laser beam is focused and can be used as a localized heat or illumination source. Second, a laser with a specific wavelength can be applied for materials to selectively absorb or transmit the laser during laser processing because the absorption spectra of materials vary from one another. Moreover, the laser penetration depth can be controlled by employing lasers of different wavelengths [10]. Third, a laser is an extremely rapid heat source, and can increase the temperature much higher than conventional heating systems in a short time. Fourth, the output laser beam can be controlled using digitized parameters. In contrast to a typical heat source for which the temperature must pass continuously from a certain temperature to the final value, the laser can induce an immediate temperature change to the designated temperature through the setting of digital parameters. This characteristic allows the facile seamless integration of a laser with other fabrication tools. Fifth, the laser process can minimize the thermal damage to the substrates. Selecting a suitable laser wavelength prevents the laser from being absorbed directly into the substrate, whereas the laser-absorbing material can be heated to a very high peak temperature and cooled quickly. This makes the laser an effective tool for processing deposited materials on heat-sensitive substrates to fabricate flexible and stretchable devices [11–15]. Sixth, lasers can have various operating modes. A continuous-wave (CW) laser emits light continuously, whereas a pulsed laser emits light for a certain duration at a specific repetition rate. Additionally, there are various pulse lasers, such as nanosecond, picosecond, and femtosecond lasers, that differ with regard to the pulse width of light, and lasers with the same pulse width can exhibit vastly different characteristics depending on the repetition rate. Lastly, the laser beam size and shape can be easily controlled by employing proper optical components. An extremely high power density can be achieved even with a low laser power because the laser power density is inversely proportional to the square of the beam diameter. On the other hand, nanomaterials exhibit unusual specific properties that differ significantly from those of bulk materials. One of the prominent phenomena occurring in nanoscale materials is melting-point depression. For example, while the melting temperature of bulk Au is > 1000 °C, that of < 3-nm Au NPs is as low as 150 °C [11]. For the aforementioned reasons, the laser process of nanomaterials has been actively developed over the past two decades as a next-generation electrode fabrication method that does not require conventional photolithographic processes, vacuum equipment, or high-temperature processes [3, 4, 12–16].

Laser digital patterning (LDP) is a photolithography-free electrode fabrication and patterning method that exploits the foregoing unique characteristics of lasers and nanomaterials. The word “digital” here indicates that the laser is controlled by digitized parameters, and a digital image or computer-aided design (CAD) image is utilized for patterning instead of a physical patterning mask. This process is often called by different names, such as laser direct writing, laser direct patterning, and laser selective patterning, but the basic concepts and principles are similar. Although LDP covers, in a broad sense, all the fabrication methods of the laser-based additive and subtractive material patterning process regardless of the materials used, the meaning of LDP in this review is limited to an electrode patterning process using thin films coated with nanomaterials. The overall procedure of the LDP process is described in Sect. 2.1. It is noted that the detailed procedure of the LDP process can be partly different depending on the material used. In the early stages, the LDP process was developed using noble metal nanoparticles (NPs) such as Au and Ag [17–19] because such materials are free from oxidation even in a nanosize configuration. Thus, the NPs just need to be fused together to form continuous conductive structures via laser irradiation. However, owing to the high prices of the noble metals—which make the process noncompetitive as a low-cost, practical electronics fabrication method—the need to use common metals for the LDP process is increasing. The most significant challenge in employing common metal NPs is that they are easily oxidized at the nanoscale and exist in oxidized forms owing to the large surface-to-volume ratio of NPs. Therefore, to fabricate conductive electrodes using common metal NPs via the LDP process, laser irradiation should sinter the NPs and simultaneously induce additional photochemical reactions, such as reduction, and defect structure modification to increase the conductivity of the electrodes. This review summarizes recent studies on the LDP process in which metal oxide NPs such as ITO, ZnO, CuO, and NiO, were exclusively utilized for fabricating conductive electrodes. The outlook of the LDP process for these materials is also discussed in the context of next-generation transparent conductors, sensors, and electronics. It is worth to note that the word “sintering” refers to solid-state fusion, whereas the word “annealing,” in a broader sense, covers melting phenomena. However, currently, the two words are used interchangeably, without a significant distinction of meaning. Therefore, in this review, these two words are quoted in the form used in the original references wherever possible.

## LDP process

### Overall procedure of LDP process of metal oxide thin films

**Fig. 1:**
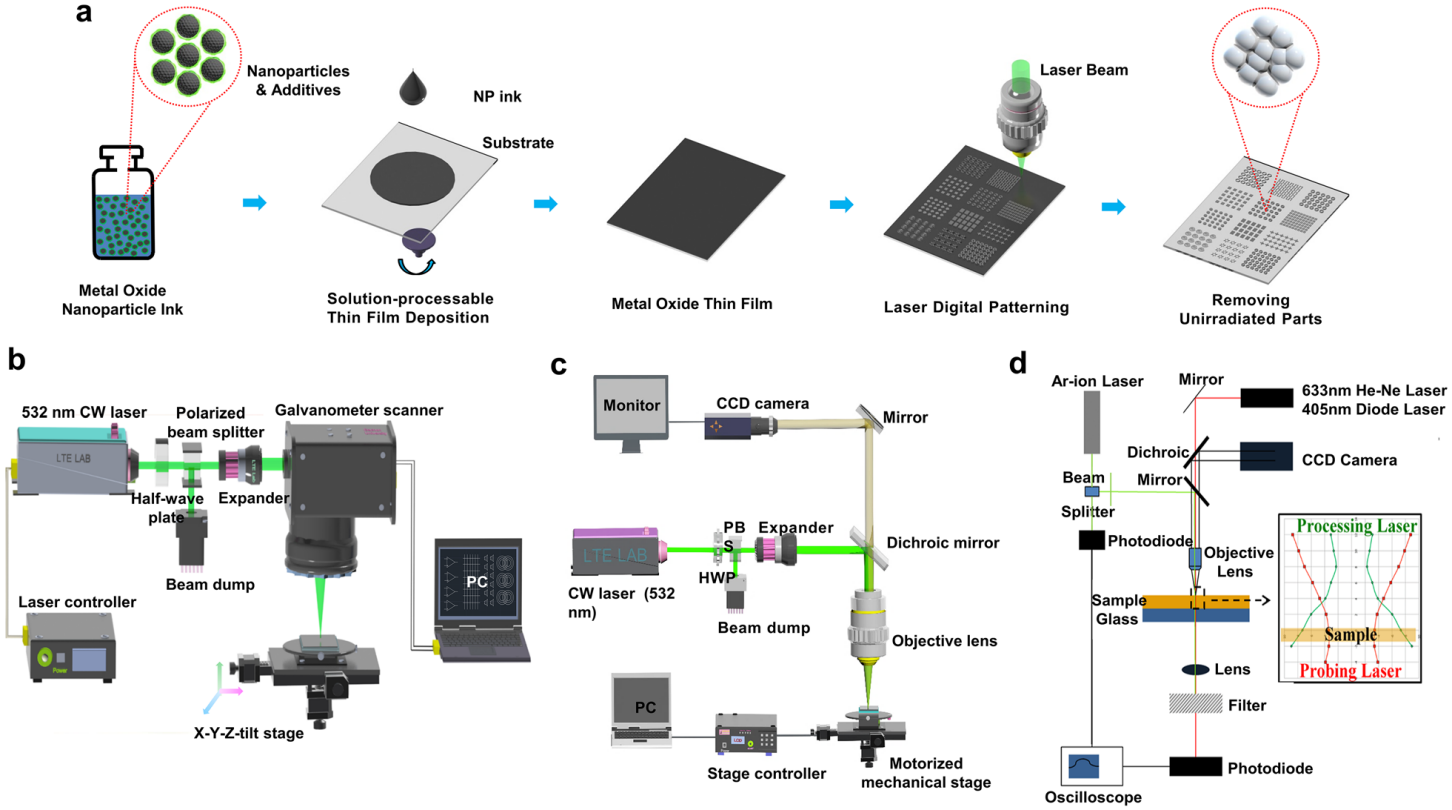
**a** Schematic of the overall procedure of the LDP process. **b** Schematic of the laser setup for moving the laser beam using a galvano mirror scanner. **c** Schematic of the laser setup for a fixed laser beam through an objective lens (Figures **b** and **c** reproduced with permission from Ref. [20]. Copyright Wiley–VCH, 2019). **d** Schematic of the laser setup for integration of a processing laser and a probing laser (Figure **d** reproduced with permission from Ref. [10]. Copyright Springer, 2015)

Figure 1a shows the overall procedure of the LDP process for the thin film deposited using metal oxide NP ink. As mentioned previously, the detailed procedure of the LDP process depends on the material used, but this review focuses on the LDP process of thin films deposited using metal oxide NP ink. First, well-dispersed NP ink is prepared. “NP ink” refers to a solution where NPs are dispersed together in a solvent. Chemical additives, such as reducing agents and dispersants, are added and mixed if required. Then, the NP ink is deposited on substrates to form thin films via solution-processable methods such as spin coating, dip coating, doctor blading, and inkjet printing. In the selective laser irradiation step, the laser irradiates a specific portion of the thin film. Upon laser absorption, the temperature of the irradiated part increases rapidly, causing sintering of the NPs, along with a reduction reaction or defect structure modification. Finally, the washing process (if required), is applied. Because the laser-irradiated parts have enhanced adhesion to the substrate, only the laser-irradiated parts remain on the substrate after the washing process. A recent study [20] indicated that the strong adhesion between the electrodes and substrates of the laser-irradiated parts originates from that fact that substrate surfaces undergo partial melting and solidification during the LDP process; thus, the melted substrate instantly forms intimate contact with the sintered electrodes through the mechanical interlocking of the partially submerged electrode with the substrate. The solvent used in the washing process is generally the same solvent used for the NP ink or a solvent that is highly miscible with the ink solvent.

The LDP process has the following unique features compared with conventional electrode patterning processes. First, the process operation is simple, convenient, and time-saving, because no vacuum apparatus is generally required, even though an additional gas environment is required in special cases. It is also convenient to integrate the LDP process with other processes to compose an in-line continuous setup. Second, changing the pattern shape is extremely simple and cost-effective compared with the conventional photolithography process, because no physical mask is required. Third, as the washing process is simple, it is not necessary to use various etching solutions (which are usually not ecofriendly). Finally, chemical reactions or photochemical reactions that cannot be induced by the conventional heating system can occur during the laser process, because the laser allows rapid heating to a high peak temperature while minimizing the thermal impact on the substrate.

### Laser setup for LDP process

In the selective laser irradiation step, the laser beam needs to move on the thin film. This can be achieved either via laser scanning using a galvano scanner (Fig. 1b) or by translating the thin film (Fig. 1c). In the former case, two galvanometer mirrors (one for each axis), control the position of the laser beam in the field of view. A telecentric lens allows the laser beam to be focused to different distances without changing the focus size. The scanning speed can reach several meters per second, and the scanning system can be easily integrated with a CAD program to generate arbitrary patterns. However, the minimum focused beam size is usually > 10 µm, even for short-wavelength laser beams. In the latter setup, the laser beam is focused through an infinity-corrected objective lens, while the thin film on a motorized mechanical stage moves under a fixed laser beam. The movement of the mechanical stage can be controlled by a CAD-integrated system. The laser beam diameter can be significantly smaller than that of the galvano mirror scanner if a large-numerical aperture objective lens is employed. Therefore, high-resolution patterning is achievable. Additionally, the latter setup is convenient for in situ monitoring of the laser processing area by a charge-coupled device camera. However, the scanning speed is limited by the performance of the mechanical stage and is significantly lower than the scanning speed of the former system. As an expanded setup, multiple lasers of different wavelengths (a processing laser and a probing laser) can be integrated together, as shown in Fig. 1d [10]. This setup allows in situ characterization of the thin film property changes during laser processing.

## LDP process of metal oxide NP thin films

### Indium-doped tin oxide (ITO)

ITO is the most widely used material for transparent conducting electrodes owing to its high conductivity and transparency [21, 22]. Most of the thin films for television/computer screens and mobile device touch panels encountered in everyday life are made of ITO thin films deposited on rigid substrates via vacuum-based methods and patterned via photolithographic processes [23, 24]. The LDP process employing ITO NPs is a promising alternative to the conventional process, as it does not require vacuum equipment and allows the production of flexible ITO thin films. Although lasers have been used as post-processing tools for vacuum-deposited ITO thin films [25–27], studies on the LDP process of ITO films prepared using NP ink have not been actively performed. Most of the studies introduced in this section focused on improving the conductivity while maintaining the transparency of ITO NP films via laser irradiation, rather than focusing on selective patterning. However, research on fabricating transparent electrodes by applying laser processes to thin films produced using ITO NP ink is considered to be worthwhile, because it is clearly differentiated from conventional processes and provides fundamental insights regarding the patterning of the material.

**Fig. 2:**
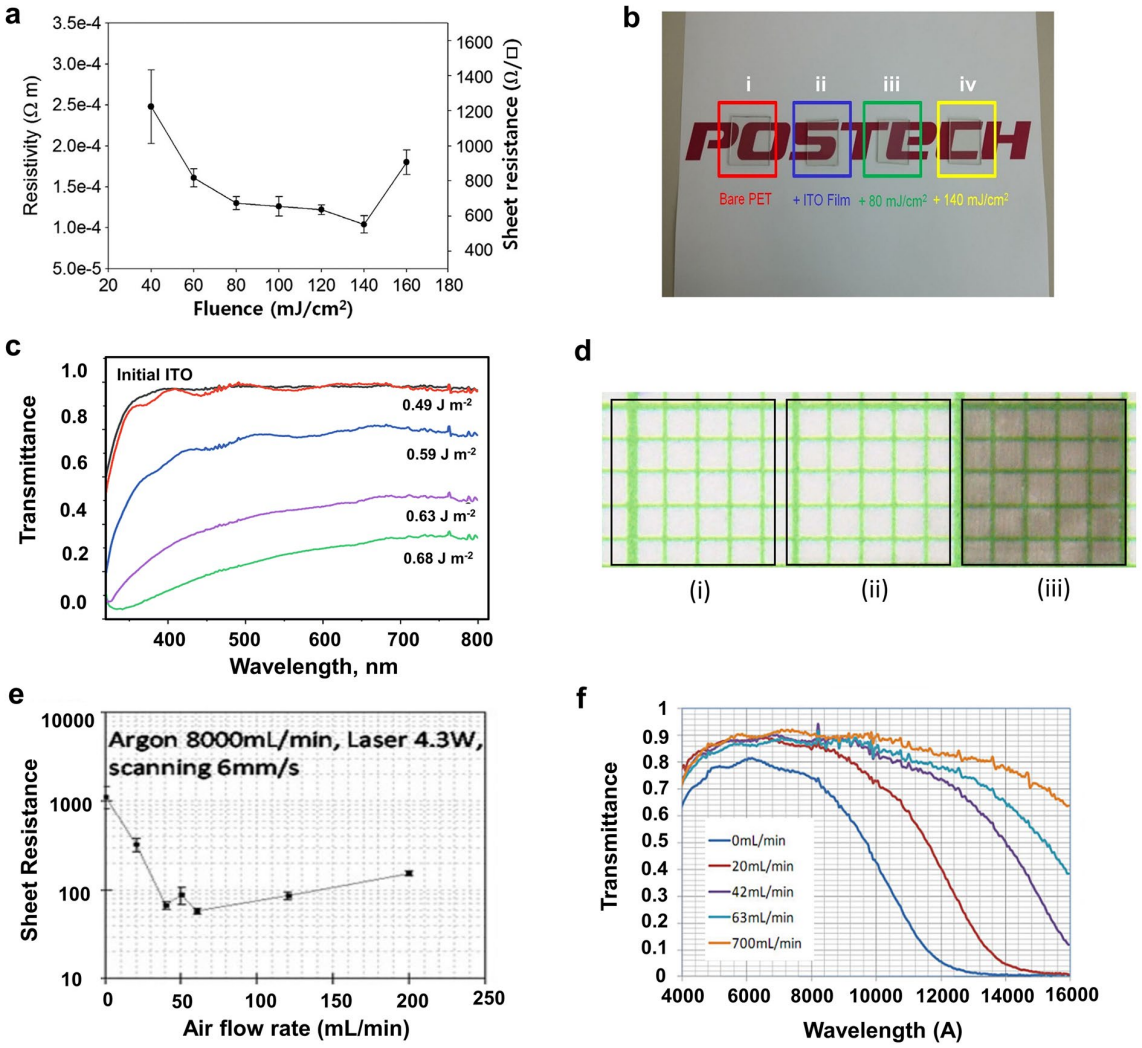
**a** Variation of the electrical resistivity (sheet resistance) of the laser-irradiated ITO film depending on the laser fluence of a single pulse. **b** Optical image to compare the transparency of thin films after the laser process (i: bare PET; ii: ITO film only; iii: ITO film after a single laser pulse at 80 mJ cm^−2^; iv: ITO film after a single laser pulse at 140 mJ cm^−2^) (**a** and **b** reproduced with the permission from Ref. [30]. Copyright Elsevier, 2015). **c** Optical transmission spectra of ITO thin films with different incident laser fluences. **d** Photographic image of a gravure-printed ITO film irradiated within the areas indicated by the black lines at different laser fluences (i: 0.49 J m^−2^; ii: 0.56 J m^−2^; iii: 0.65 Jm^−2^). Paper with millimeter squares was used as a background (Figures **c** and **d** reproduced with permission from Ref. [33], Copyright Nature Publishing Group, 2019). **e** Dependence of the sheet resistance of the laser-annealed ITO NP thin film on air–Ar mixed background gas in a quartz enclosure. **f** Transmittance data for a laser-annealed ITO NP thin film measured under different air flows with a fixed Ar flow at 8000 mL/min (**e** and **f** reproduced with permission from Ref. [29]. Copyright Springer, 2011)

The carrier density of ITO is determined by active Sn dopants and O vacancies, whose generation reaction depends on the temperature [28, 29]. Therefore, the temperature increase in ITO NPs induced by the laser not only sinters the NPs, fusing them together, but also induces carrier density changes. UV lasers are effective for ITO processing because their photon energy is larger than the bandgap of ITO (3.5–4.3 eV); thus, interband absorption occurs. Park et al. used nanosecond KrF excimer laser irradiation (λ = 248 nm, pulse width = 25 ns) to enhance the electrical conductivity of an ITO NP thin film on a heat-sensitive polyethylene terephthalate (PET) substrate [30]. A 200-nm-thick ITO thin film with a high sheet resistance (~ 900 kΩ sq^−1^) was fabricated on PET by spin-coating an ethanol-based ITO NP ink. Subsequent single-pulse laser irradiation of fluence in the range of 80–160 mJ cm^−2^ fused ITO NPs to form crystallized grains. Consequently, the sheet resistance was reduced to approximately 500 Ω sq^−1^ (Fig. 2a). Even though the transmittance of the ITO film decreased by up to 24% from the maximum value, the optical transparency level of the film was still comparable to that of the sputtered film (Fig. 2b). Additionally, infrared (IR) lasers can be used for the laser processing of ITO thin films, because free carriers that exist in the film allow the intraband absorption of the IR laser in ITO [31]. KÖniger et al. reported that CO_2_ laser irradiation (λ = 10.6 µm) improved the electrical conductivity of ITO NP thin films coated on a PET substrate [32]. The sheet resistance of the laser processed ITO thin film was reduced by a factor of 30 compared with that of an as-prepared one, while its transparency exhibited only a slight decrease of approximately 5% and remained above 75% at the laser fluence of 40 kJ m^−2^. Furthermore, the electrical conductivity exhibited high stability under bending deformation owing to its porous structures [32]. Serkov et al. used a combination of gravure printing of ITO NP ink and a laser sintering process to generate highly conductive films without affecting the PET substrate [33]. A laser (λ = 1750 nm, pulse width = 6 ps, pulse repetition rate = 40 MHz) was applied to the ITO film with fluence variations (0.35–0.8 J m^−2^). The sheet resistance decreased significantly from 6 kΩ sq^−1^ at a fluence of 0.35 J m^−2^ to 0.1 kΩ sq^−1^ at a fluence of 0.7 J m^−2^, which was attributed to the laser-induced removal of the dielectric 3-methacryloxypropyltrimethoxysilane (MPTS) binder in the ink film and the sintering of the NPs. However, the transparency of the irradiated film became more opaque with an increase in the laser fluence owing to the increased amount of metallic phases after the laser process, as shown in Fig. 2c, d [33]. By optimizing the laser parameters, a low sheet resistance of 0.5 kΩ sq^−1^ was achieved, while the transmittance exhibited a slight variation, at a laser fluence of 0.49 J m^−2^.

Pan et al. used a near-IR CW fiber laser (λ = 1.5 µm) to sinter ITO NPs and achieved a resistivity of 1.3 × 10^−3^ Ω cm, which is 4–5 times higher than that of commercially available sputtered ITO films [29]. They employed a cylindrical lens to form an elliptical beam to increase the processing area. By introducing Ar and air mixtures as background gases, the dependencies of the ITO sheet resistance and transmittance on the gas composition (Fig. 2e, f), as well as the mechanism for controlling the carrier density associated with defect kinetics in the ITO thin film, were investigated.

### ZnO

ZnO is one of the major semiconductors in the field of nanoscience and nanoengineering and is used for various electronics, photonics, optics, and sensors for the following reasons: (1) ZnO is an abundant, nontoxic, and low-cost material; (2) ZnO nanostructures can be synthesized via a simple method at a low temperature; and (3) the optoelectronic properties of ZnO NP films can be easily tuned through doping with various dopants. In particular, doped ZnO and undoped ZnO have long been studied as substitutes for ITO, and related research is still underway. However, few studies have been performed on the laser processing of solution-processed NP-based ZnO thin films, whereas studies on the laser annealing of ZnO thin films deposited through sputtering, evaporation, sol–gel processes, and pulsed laser deposition [34–41] have been actively reported. The researches presented below deal with ZnO thin films deposited using ZnO NPs but the final washing process illustrated in Fig. 1 was not applied. However, it is worth introducing the researches in this chapter, because the fundamental concept and purpose of the laser process match the scope of this review in that laser irradiation is applied to the NPs and induces additional photochemical reactions simultaneously to control the conductivity of the electrodes.

**Fig. 3:**
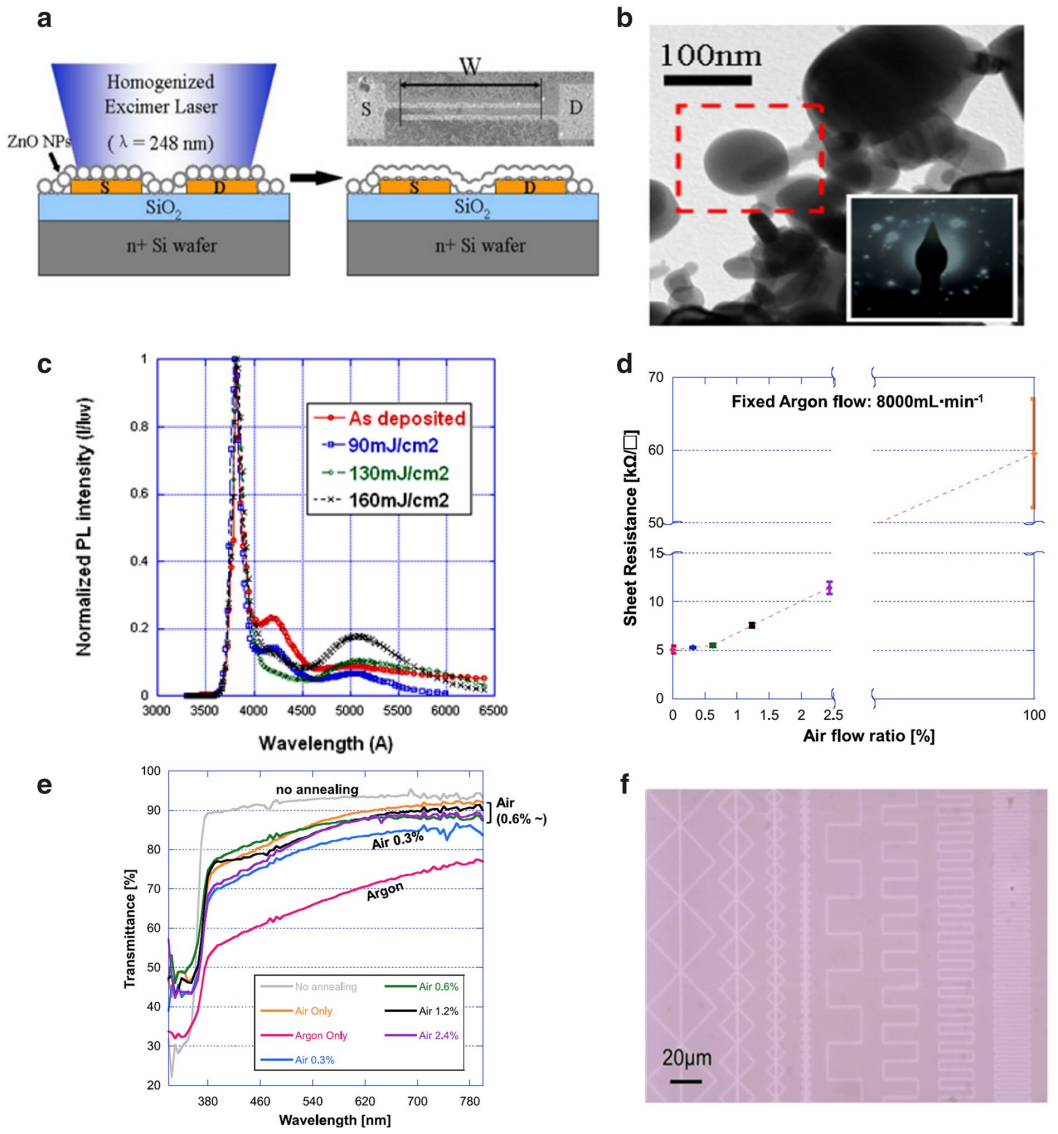
**a** Schematic side view of the excimer laser annealing process and the ZnO NP field-effect transistor structure. S and D indicate the source and drain electrodes, respectively. The top right inset presents a top-view SEM image of transistor electrodes with a layer of deposited ZnO NPs. **b** Transmission electron microscopy (TEM) image of the ZnO NP film after excimer laser irradiation. The inset shows the diffraction pattern for the marked region. **c** Room-temperature photoluminescence spectra of the ZnO thin film with and without laser annealing (**a**–**c** reproduced with permission from Ref. [42]. Copyright Springer, 2009). **d** Sheet resistance of the ZnO thin film depending on the background gas during laser annealing. **e** Transmittance of ZnO thin films annealed in background gases with different compositions. **f** Optical microscope image of 1.5-µm-line width patterns generated on the ZnO thin film (**d**–**f** reproduced with permission from Ref. [44]. Copyright Springer, 2012)

For the material to absorb the laser via interband absorption, lasers with photon energies equal to or higher than the bandgap of the material are required. Because the bandgap of ZnO is ~ 3.3 eV, ultraviolet (UV) lasers with wavelengths shorter than 375 nm ($\frac{{1240 \;{\text{eV }}\;{\text{nm}}}}{{3.3\; {\text{eV}}}} \approx 375 \;{\text{nm}}$) can be utilized to process ZnO thin films. Pan et al. reported the laser sintering of solution-deposited ZnO NPs to produce an active layer of a high-performance n-type field-effect transistor (FET) [42]. The ZnO NP ink was prepared by dispersing commercial NPs (< 100 nm) in ethanol and deposited via spin coating on the pre-patterned metal contacts, which were defined on an SiO_2_ gate dielectric formed on a highly doped n-type Si wafer. The ZnO layer was then irradiated by a single pulse of a flat-top excimer laser beam (λ = 248 nm, pulse width = 20 ns) at a fluence of 160 mJ cm^−2^ (Fig. 3a). Upon laser irradiation under ambient conditions, the NPs melted to form polycrystalline, rounded, and smooth agglomerations (Fig. 3b). Photoluminescence measurements (Fig. 3c) confirmed that the high photon energy of the laser broke chemical bonds, created O vacancies, and removed excess O, reducing the electrical resistance of the ZnO layer by three orders of magnitude and increasing the mobility and on/off ratio of the ZnO FET by three to four orders of magnitude. It is noted that only single-pulse laser irradiation resulted in a similar effect to ~ 700 °C furnace annealing [43] on the mobility of the ZnO FET. In another study, Lee et al. used a high-repetition UV picosecond laser (λ** = **355 nm, pulse width = 12 ps, pulse repetition rate = 80 MHz) to increase the electrical conductivity of a transparent ZnO thin film that was deposited with well-dispersed, high-concentration ZnO NP ink containing very small (~ 6 nm) NPs [44]. The ZnO NPs synthesized in the methanol-based solution were re-dispersed in a longer-chain alcohol (1-pentanol) to prevent agglomeration of the NPs. Upon laser irradiation under ambient conditions, the temperature of the ZnO NP thin film increased sufficiently to break the Zn–O bonds and form additional O vacancies. Simultaneously, the NPs were fused to each other, creating continuous current paths, which increased the conductivity. Laser sintering with an Ar flow further enhanced the conductivity of the ZnO thin film (although the transmittance of the resulting thin film was reduced), as it promoted a net increase in O vacancies by preventing the oxidation of ZnO by ambient air (Fig. 3d, e). Adjusting the ratio of air to Ar as background gases can produce a highly conductive ZnO thin film while maintaining high transparency. The lowest resistivity achieved with the undoped ZnO thin film in this study was 4.75 × 10^−2^ Ω cm, which was five orders of magnitude lower than that of a furnace-annealed ZnO film [45]. Because the unirradiated parts of the ZnO thin film were almost insulators, arbitrary conductive ZnO patterns could be generated directly on the substrate via laser processing (Fig. 3f).

As mentioned previously, the optoelectronic properties of ZnO films can be easily tuned via doping with various dopants, such as Al [46], Ga [47, 48], and Li [49]. Among the dopants, laser annealing of Al-doped ZnO (AZO) thin films prepared via vacuum-based deposition or sol–gel processes have been extensively investigated, and the optoelectronic properties of the resulting films are comparable to those of ITO films. For instance, Hamali et al. used excimer laser irradiation (λ = 248 nm, pulse width = 25 ns) to enhance the conductivity of a sputtered AZO film [50]. After laser processing at a fluence of 125 mJ cm^−2^, the resistivity of the thin film was reduced from 1 × 10^−3^ to 5 × 10^−4^ Ω cm, while the average visible transmission was increased from 85 to 90%. In contrast to the case of undoped ZnO thin films, lasers with a wide wavelength range (from UV to IR) can be used for the processing of doped ZnO films, owing to the absorption of free carriers of the dopants and the modification of the band structure due to doping. For example, Hsiao et al. used an 808-nm CW laser [51], Xu et al. used a 1064-nm CW laser [52], and Huang et al. used a 532-nm nanosecond laser [53] for AZO annealing. However, thus far, no LDP process for a doped ZnO thin film prepared using NP ink has been reported, to the best of our knowledge.

**Fig. 4:**
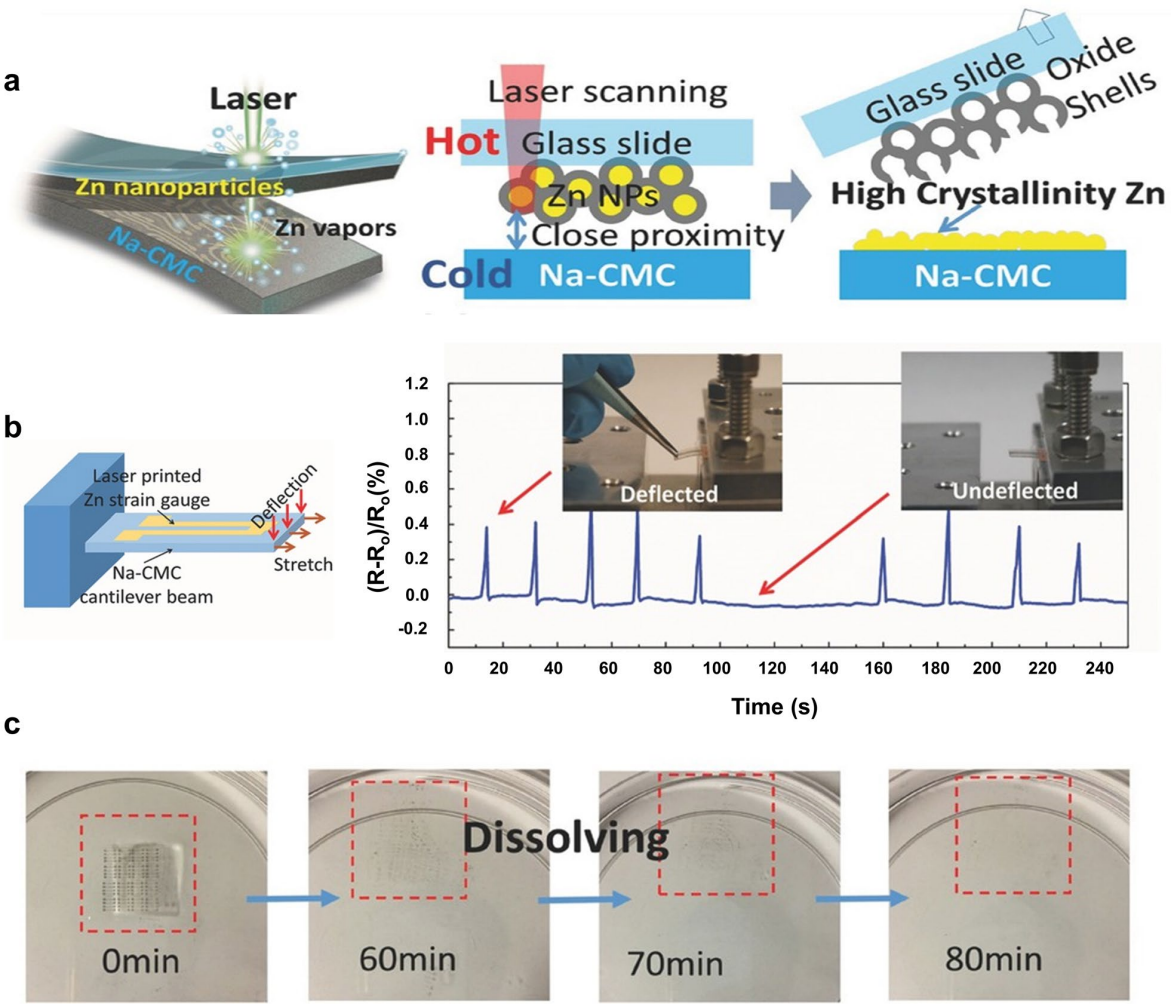
**a** Technical illustration and schematics of the laser printing process to generate Zn electrodes on Na-CMC. **b** Schematic of the laser-printed resistive Zn strain gauge. Response of the strain gauge under deflection. **c** Photograph of the dissolution process of Zn patterns on the Na-CMC substrate in distilled water (**a**–**c** reproduced with permission from Ref. [55]. Copyright Wiley–VCH 2017)

More recently, several studies indicated that the LDP of ZnO thin films allows the fabrication of transient electronic devices that can disappear completely or partly upon exposure to water at prescribed rates or within a prescribed time period [54–56]. Shou et al. reported a low-cost laser process for ZnO NP films to fabricate Zn conductors on a bioresorbable sodium carboxymethyl cellulose (Na-CMC) substrate [55]. Although Zn NPs were used as starting materials for the laser process in this study, oxide-shell layers were formed naturally on the Zn NPs, yielding ZnO, which made the sintering process difficult. The authors prepared a Zn NP-coated glass slide, which was gently placed on a Na-CMC substrate, and applied a 1065-nm CW laser beam driven by a galvano scanner through the top glass slide. The laser irradiation removed the oxide shell layers of the Zn NPs and then transferred arbitrary electrode features onto the receiving Na-CMC substrate (Fig. 4a). The resulting resistivity of the laser-irradiated ZnO electrode was approximately 0.9 × 10^−4^ Ω cm, which is 15 times higher than that of bulk Zn (6 × 10^−6^ Ω cm). The usefulness of the Zn electrodes was demonstrated by fabricating a strain gauge that could detect both stretching and deflection (Fig. 4b). The Zn electrodes on the substrate dissolved completely in water within 100 min (Fig. 4c).

### CuO

Cu has received considerable attention as an alternative to ITO and noble metals (Au, Ag) owing to its remarkably low cost and superior electrical conductivity [57–62]. Cu has a low electrical resistivity (1.68 μΩ cm), which is similar to that of Ag (1.58 μΩ cm) and lower than that of Au (2.21 μΩ cm), but is 100 times less expensive because it is 1000 times more abundant [63]. Moreover, CuO nanostructures with various morphologies and dimensions can be easily synthesized using numerous techniques, such as chemical solutions [64–66], the hydrothermal method [67–69], and the sol–gel method [70–72]. However, nanoscale Cu materials are easily oxidized, becoming nonstoichiometric CuO_x_ (e.g., Cu_2_O, CuO), under ambient conditions because of their low oxidation potential energy. Therefore, many studies have been conducted to enable the use of Cu nanomaterials by reducing the synthesized CuO using microwaves [73], atmospheric-pressure glow (APG) discharge [74], and intense pulsed light (IPL) [75, 76]. However, there are limitations; e.g., microwave processing requires a high temperature and a large amount of electricity; vacuum conditions and a high-temperature environment are required for APG discharge-based processing; the IPL method requires an additional photomask to create a fixed electrode pattern. LDP process-induced reductive sintering (LRS) of the CuO NP film with the aid of reducing agents was proposed as a new alternative for fabricating Cu patterns without high-temperature, high-vacuum environments or photolithographic processes.

**Fig. 5:**
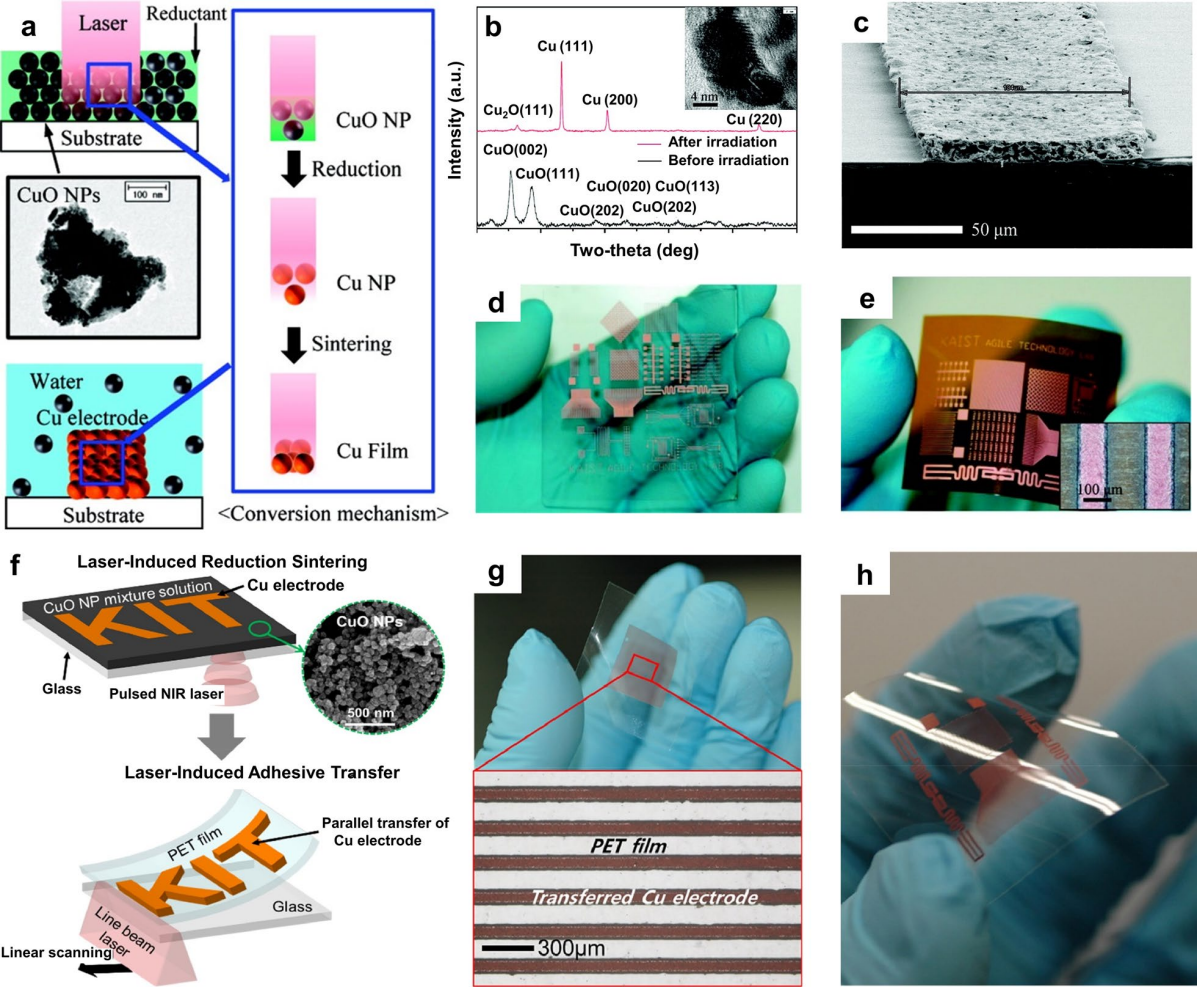
**a** Schematic of the proposed process for the conversion of CuO NPs into a Cu film via photochemical reduction and photothermal agglomeration. **b** XRD analysis results obtained before and after laser irradiation; (inset) TEM image of a Cu film processed using a pulsed laser. **c** Cross-sectional SEM image of the Cu electrode. **d, e** Photograph of Cu electrode patterns on a glass substrate and a PI substrate, respectively (**a**–**e** reproduced with permission from Ref. [77]. Copyright American Chemical Society 2011). **f** Schematic of the fabrication procedures for laser-induced reductive sintering and laser-induced adhesive transfer. **g** Photograph and microscope image of Cu NP electrodes transferred to a PET receiver film at a laser power of 2 W and a laser scanning speed of 2000 mm min^−1^. **h** Photograph of arbitrary Cu electrode patterns transferred to the PET film (**f**–**h** reproduced with permission from Ref. [81]. Copyright Elsevier, 2018)

Kang et al. reported a simple and low-cost fabrication route to generate Cu electrode patterns using the LRS process [77]. CuO ink was prepared by dispersing CuO NPs into a polyvinylpyrrolidone (PVP) and ethylene glycol (EG) mixture and spin-coated onto a UV–ozone-treated glass or flexible polyimide (PI) substrate. Because of the narrow bandgap of CuO (1.1–1.2 eV), 1070-nm laser irradiation (either CW or pulsed mode) was absorbed well by the thin CuO film through interband absorption. During the laser process, EG generated acetaldehyde via dehydration, and CuO NPs were converted into Cu NPs (Fig. 5a). Reduction from CuO to Cu was confirmed by a color change (from dark to reddish) and by X-ray diffraction (XRD) measurements (Fig. 5b) and indicated the low resistivity of the Cu electrode pattern (31 μΩ cm) (Fig. 5c–e). Lee et al. examined the reduction tendency of CuO under pulsed laser irradiation (λ = 1070 nm, pulse repetition rate = 1 MHz) after dissolving PVP as a reducing agent in different solvents, such as isopropyl alcohol, ethanol, and 1-butanol [78]. Upon laser irradiation, the PVP was decomposed into amorphous C and other materials by thermal energy in a hydrate solvent. Among the other generated materials, carboxylic acid reduced CuO to Cu, and residues such as amorphous C, hydrogen oxide, and carbon dioxide were removed by the washing process or evaporated during the laser processing. The as-prepared thin film composed of exothermic solvents after the LRS process exhibited a better morphology and higher conductivity than other films composed of endothermic solvents. Additionally, a larger amount of conductive electrodes could be realized with higher molar ratios and larger amounts of PVP in the CuO ink. By optimizing the PVP concentrations and solvents, a Cu electrode with a low specific resistance of 13 µΩ cm was achieved. Mizoshiri et al. fabricated Cu and Cu/Cu_2_O electrodes by controlling the scanning speed (500–1000 µm s^−1^) and pulse energy (0.36–1.2 nJ) during the LRS process using a femtosecond laser (λ = 780 nm, pulse width = 120 fs, pulse repetition rate = 80 MHz) [79, 80]. The electrical resistance of the Cu electrodes exhibited metal-like electrical conductivity behavior with a positive temperature coefficient of resistance, whereas that of the Cu_2_O electrodes exhibited semiconductor-like behavior with a negative temperature coefficient of resistance. These properties can be used to fabricate temperature-sensing materials.

Processing on a material substrate having a low glass transition temperature (T_g_ ≈ 80 °C), such as PET, is another challenge. Back et al. developed a new approach for Cu electrode patterning on PET substrates that combined a laser-induced adhesive transfer process with the LRS technique (Fig. 5f) [81]. First, a Cu electrode was fabricated on a glass donor by inducing reduction and agglomeration of the CuO NP film via irradiation with a focused beam of a pulsed fiber laser (λ = 1070 nm, pulse repetition rate = 300 kHz). Subsequently, the Cu electrode formed in the previous step was transferred to the PET substrate via 808-nm CW laser-induced adhesive transfer between the PET film and the donor surface (Fig. 5g, h) [81]. The resulting Cu electrodes exhibited a low resistivity of 30 μΩ cm, with strong adhesion to the PET substrate.

**Fig. 6:**
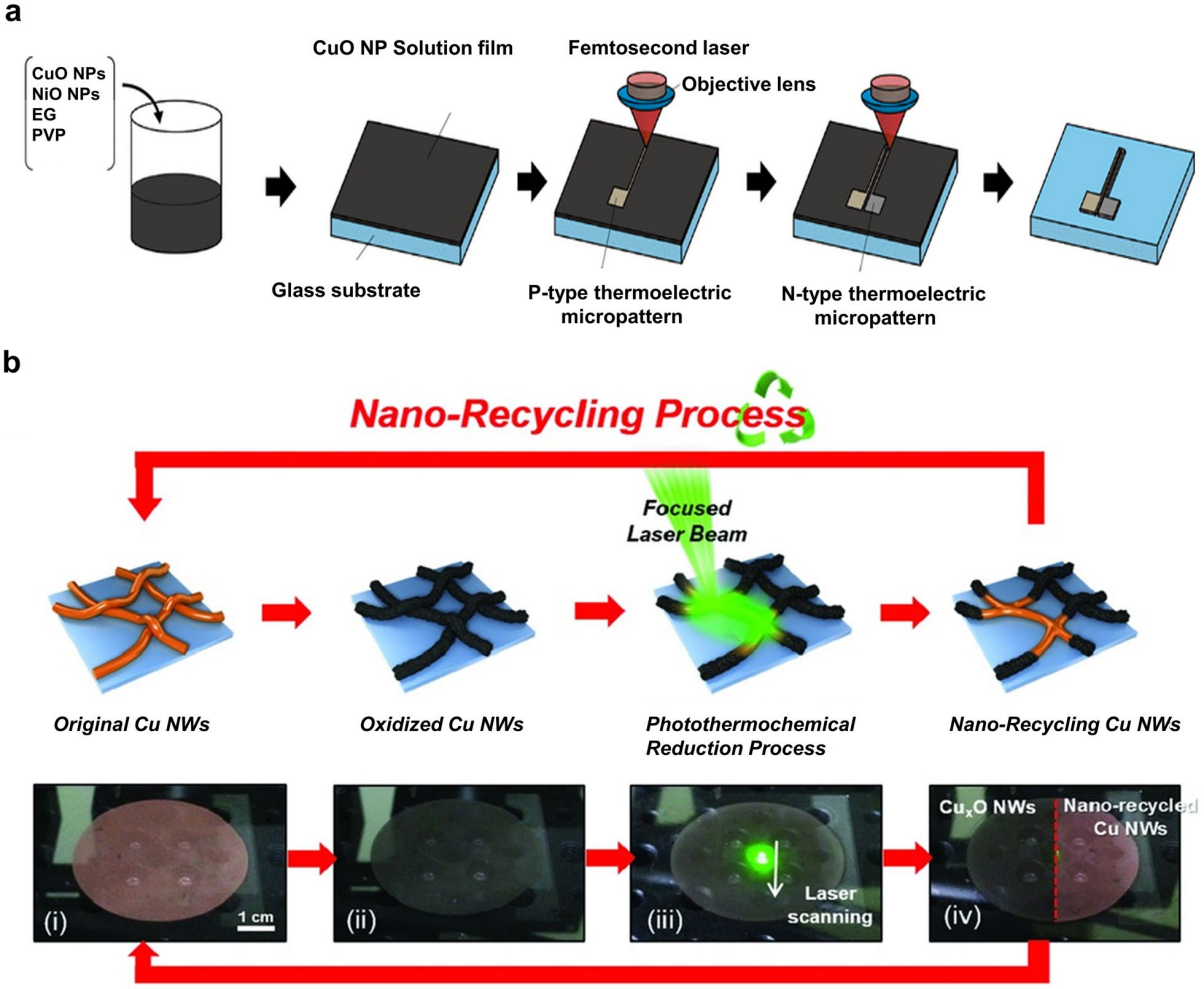
**a** Selective fabrication of p- and n-type thermoelectric micropatterns via the reduction of CuO–NiO mixed NPs using femtosecond laser pulses. (Reproduced with permission from Ref. [83]. Copyright Springer, 2017). **b** Schematic of the nano-recycling process flow, with optical photographs corresponding to each stage of nano-recycling (Figures reproduced with permission from Ref. [85]. Copyright Wiley–VCH, 2015)

More recent studies indicated that the implementation of the LRS process for a CuO-based mixture containing NiO [82, 83] and graphene oxide (GO) [84] allows the fabrication of multi-element composites with various functional properties. Mizoshiri et al. fabricated p- and n-type thermoelectric micropatterns using the LRS process of CuO/NiO NP films (Fig. 6a) [83]. The CuO/NiO NP ink was prepared by mixing PVP, CuO NPs, NiO NPs, and EG and was spin-coated onto the glass substrate. By controlling the scanning speed of the focused femtosecond laser (λ = 780 nm, pulse repetition rate = 80 MHz) on the CuO/NiO NP film, Cu_2_O/NiO or Cu/Ni electrodes could be fabricated. Cu_2_O/NiO electrodes fabricated with scanning speeds of 1 and 20 mm s^−1^ exhibited p- and n-type thermoelectric properties, respectively. The selective direct writing process for p-/n-type thermoelectric materials indicated the potential for application to various thermoelectric-type sensors. Watanabe et al. synthesized a reduced GO (rGO)/Cu hybrid structure by applying the LRS method to a water-dispersion mixture of GO and CuO nanorods (NR) [84]. GO was used as a reducing agent and as a gel-like binder to form a coating film. In this study, the well-known reaction involving the reduction of CuO with C via heat treatment was exploited. During the laser irradiation, the oxidized structure of GO was easily changed into rGO, while the CuO NRs were reduced to Cu. The hybrid rGO/Cu and GO/CuO structures were used for an IR photosensor. The concept of converting CuO_x_ nanostructures into Cu through the LRS process has been extended to the repetitive reuse of nanomaterials (i.e., nano-recycling) via the photothermochemical reduction process [85]. Applying 532-nm CW laser irradiation to the oxidized Cu nanowire (NW) electrode layer with additional EG on the layer induced dehydration of EG, generating acetaldehyde, which reduced CuO to Cu, with diacetyl and water as byproducts (Fig. 6b). The recycled Cu electrodes exhibited excellent conductivity, with increased resistance to the external environment owing to the enhanced NW junctions. The authors confirmed that the recycling process can be applied repeatedly.

### NiO

**Fig. 7:**
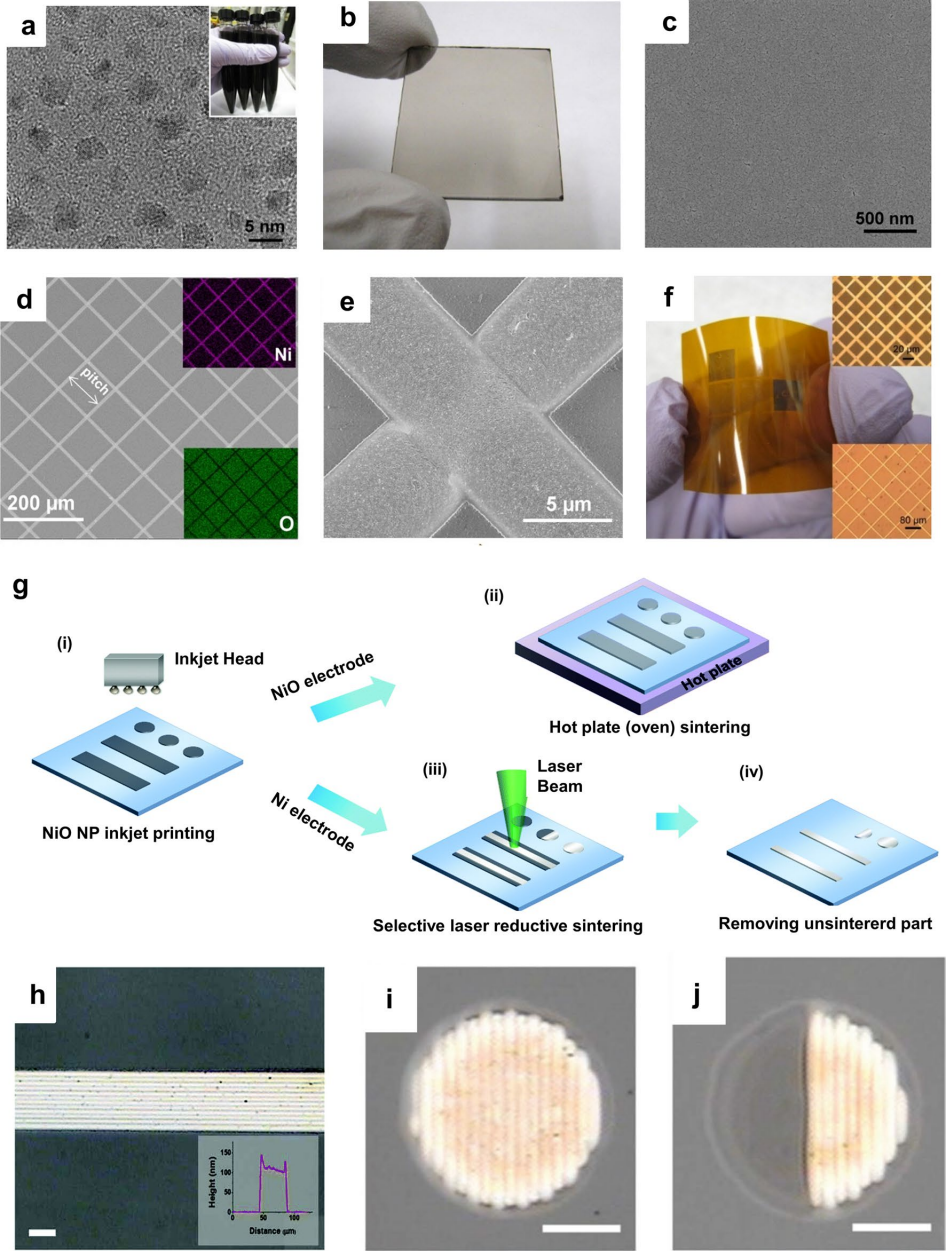
**a** TEM image of synthesized NiO_x_ NPs. **b** Spin-coated NiO_x_ thin film on a glass substrate. **c** SEM image of the surface of the NiO_x_ film shown in **b**. **d**, **e** Top-view SEM images of mesh-type electrodes with different magnifications. The insets in **d** show elemental mapping images obtained via energy-dispersive X-ray spectroscopy. **f** Mesh-type Ni electrodes with different pitches on a PI substrate. The top and bottom insets show bright-field microscopic images of the mesh patterns generated with 20- and 80-μm pitches, respectively (**a**–**f** reproduced with permission from Ref. [105]. Copyright American Chemical Society 2014). **g** Schematics of the overall experimental procedures of the NiO NP inkjet printing and sintering processes; (i) NiO inkjet printing, (ii) hotplate (oven) sintering for NiO electrodes, (iii) selective laser reductive sintering for Ni electrodes, and (iv) removal of un-sintered parts via washing. **h** Photographs of Ni patterns formed by laser reductive sintering after washing to remove non-irradiated parts. The inset shows a cross-sectional profile of the Ni pattern. **i, j** Single dot of Ni and Ni/NiO hybrid electrode, respectively, obtained via laser reductive sintering. The scale bars indicate 20 μm (**g**–**j** reproduced with permission from Ref. [108]. Copyright Royal Society of Chemistry, 2016)

Even though Cu has attracted considerable attention as an alternative to Ag owing to its cost-effectiveness and superior conductivity, Cu electrodes are vulnerable to oxidation under high-temperature and high-humidity conditions and are susceptible to corrosion in aggressive media. In contrast to Cu, Ni has a high corrosion resistance and heat resistance, along with a color similar to that of Ag; hence, it is widely used for plating metals [86–88], composite materials [89–91], and catalysts in electrochemical devices [92, 93]. On the other hand, NiO can be used as a semiconductor electrode for various applications, such as resistive switching memory [94, 95], batteries [96, 97], sensors [98, 99], solar cells [100, 101], and organic light-emitting devices [102–104]. Research on the fabrication of Ni electrodes using NPs was conducted later than research on the aforementioned materials. Similar to the case of Cu, Ni nanostructures are easily oxidized, resulting in oxide forms. Thus, in the fabrication of Ni electrodes using NiO NPs via the laser process, reduction and sintering should occur simultaneously. Lee et al. employed LDP for high-resolution Ni electrode fabrication on glass and PI substrates using solution-processed NiO_x_ NP thin films, as shown in Fig. 7a–f [105]. The nonstoichiometric NiO_x_ NPs employed in this process had a narrower bandgap (~ 2.28 eV) [106] than those used in other studies (> 3.4 eV) [93, 107] because of the higher density of oxygen vacancies; thus, 514.5-nm laser irradiation was easily absorbed by the NP thin film. Well-dispersed NP ink containing extremely small and uniform-sized NiO_x_ NPs resulted in an extremely smooth and uniform surface morphology. The NiO_x_ NP ink used in the study did not include EG or PVP; these substances played critical roles as reducing agents for CuO in other studies [77–79, 85]. Therefore, the reduction of the NiO_x_ NPs occurred via a different route [106]. (1) Laser irradiation induced a rapid temperature increase of the NiO_x_ NP film, resulting in the decomposition of the residual toluene solvent in the film, as well as interband excitation of the electrons in the NiO_x_ NP. (2) Protons generated by toluene decomposition and free electrons donated by the NPs contributed to the reduction process. (3) The reduced NPs were fused together, completing the sintering process. The role of the residual solvent in the reduction reaction was verified, as the reduction did not occur in the case of laser irradiation of the completely dried thin film. The lowest resistivity of the Ni electrode was calculated to be approximately 63 μΩ cm, which is approximately 9 times higher than that of bulk Ni (6.93 μΩ cm at room temperature), probably owing to the nanopores created in the sintered electrodes, re-oxidation, or incomplete reduction. Later, Rho et al. used a combination of inkjet printing and the LRS process for Ni electrode fabrication, as shown in Fig. 7g–j [108]. Instead of spin-coating the NiO NPs, the authors employed a drop-on-demand inkjet printing system to generate arbitrary NiO_x_ dot arrays and line patterns on specific parts of the substrate. Inkjet printing is beneficial for minimizing NP ink waste and unwanted contamination of the substrate. Subsequent laser reductive sintering applied to the patterns successfully converted the NiO_x_ into conductive Ni electrodes. Furthermore, a half-NiO/half-Ni electrode structure was obtained by applying oven sintering and the laser process consecutively.

**Fig. 8:**
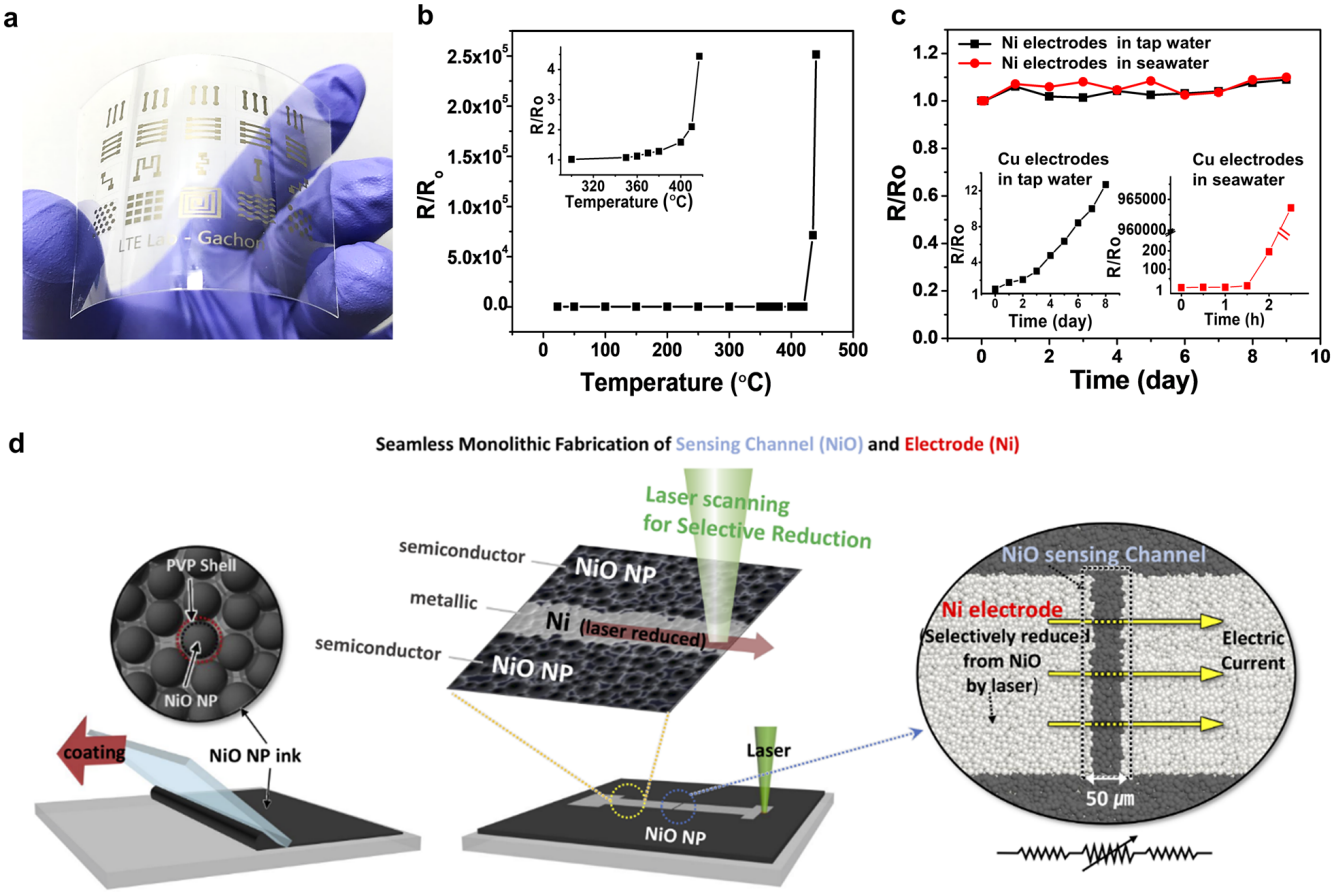
**a** Customized Ni conductor patterns on a PET substrate. **b** Relative resistance variation (R/R_o_) with an increase in the temperature from room temperature to 440 °C. The inset shows a magnified view in the temperature range of 300–420 °C. **c** Measured R/R_o_ of Ni electrodes in tap water and seawater. The insets show the measured R/R_o_ values of the Cu electrodes in tap water (left) and seawater (right) (**a**–**c** reproduced with permission from Ref. [20]. Copyright Wiley–VCH, 2019). **d** Schematic of the monolithic LRS process. NiO NP ink is coated on a substrate via the doctor blading technique. Selective laser irradiation of the dried NiO layer using a computer-aided galvano-mirror system and a monolithic Ni-NiO–Ni structure having a NiO-channel (several tens of micrometers wide) formed via the simple hatching technique (**d** reproduced with permission from Ref. [109]. Copyright Wiley–VCH, 2020)

Recently, Nam et al. developed an LRS process to fabricate Ni electrodes by employing another type of NiO_x_ NP ink that can be produced on a large scale. To produce NiO_x_ NPs, a large amount of Ni(OH)_2_ was easily prepared via the chemical precipitation method followed by calcination. NiO_x_ NPs were dispersed in 1-pentanol with PVP and cetyltrimethylammonium bromide (CTAB), which functioned as a reducing agent and a dispersant. Subsequent LDP on the spin-coated NiO_x_ NP film generated Ni electrode patterns even on PET substrates (Fig. 8a) [20]. Upon CW laser irradiation (λ = 532 nm), PVP was decomposed, yielding carboxylic acid, which reduced the NiO_x_ NPs to Ni NPs. Because the reduction reaction was observed even on a completely dried NiO_x_ NP thin film, it was concluded that the residual solvent was not an essential component for the reduction in this case. Compared with Cu electrodes, Ni electrodes exhibited distinct properties, such as excellent oxidation resistance (up to 400 °C) and high corrosion resistance in both water and seawater, as shown in Fig. 8b, c [20]. The lowest resistivity of the Ni electrode was approximately 98.9 μΩ cm, which is sufficiently low to apply the electrode to a flexible resistive touch screen panel or a transparent heater. For example, the mesh-patterned Ni panels on PET exhibited a high transparency (*T* = 84%) and low sheet resistance (*R*_s_ = 53 Ω sq^−1^). Furthermore, high mechanical flexibility and electrical stability of the Ni electrodes on PET were confirmed by various tests such as ultrasonic-bath, tape-pull, bending/twisting, and cyclic bending tests. More recently, a monolithic seamless Ni-NiO–Ni heterostructure was fabricated via the LDP process (Fig. 8d) [109]. In that study, 532-nm CW laser irradiation not only promoted photothermochemical reduction and the sintering of the NiO NPs to pattern the Ni electrodes but also induced reductive rapid thermal annealing of NiO NPs located between two Ni electrodes, forming a NiO sensing channel. Because the Ni electrodes and the NiO sensing channel were formed on a single layer, they formed a monolithic contact without any contact resistance, and no alignment process was required. Furthermore, the low-heat budget of the laser process allowed fabrication of the flexible structure even on a 25-µm-thick (ultrathin) PET substrate. Importantly, the unique thermal activation mechanism originating from the laser process enhanced the temperature sensitivity. The LDP process of NiO NPs has also been employed to produce Ni-based composite microstructures. Mizoshiri et al. used a high-repetition rate femtosecond laser (λ = 780 nm, pulse width = 120 fs, repetition rate = 80 MHz) to fabricate Ni-Cu/NiO-Cu_2_O micropatterns using a mixture of NiO and CuO NPs [82, 83] and fabricate Ni–Cr composites using NiO/Cr mixed NPs [110]. In these studies, the authors manipulated the laser scanning speed to control the chemical compositions of the composites.

## Conclusions and outlook

Recent studies on the LDP process for metal oxide nanomaterials such as ITO, ZnO, CuO, and NiO in the context of fabricating conductive electrodes were reviewed. The reviewed studies are summarized in Table 1. It is noted that the transmittance value is included in Table 1 when a transparent panel fabrication is demonstrated in the study. The LDP process for metal oxides provides a facile and versatile fabrication route compared with the conventional photolithography process, which is complex, expensive, and time-consuming. By exploiting the unique characteristics of lasers, it is possible to induce physical, chemical, and electrical changes in the material properties on heat-sensitive substrates that cannot be achieved using ordinary heat and light sources. Additionally, because the process is localized and selective, arbitrary electrode patterns can be generated in the desired form without using a photomask, and the pattern design can be modified by simply changing the drawing in the CAD system linked to the laser scanner. More importantly, the laser-induced photochemical or photothermochemical reaction allows the use of metal oxides instead of expensive noble metals as starting materials for the production of conductive electrodes, which is advantageous with regard to handling, storage, and mass production.

**Table 1:** Summary of studies on the laser digital patterning process using metal oxide nanomaterials

Metal oxide	Solvent for NP ink	Laser	Substrate	Electrical properties	Transmittance @ 550 nm	Refs.
ITO	Ethanol	ns, 248 nm	PET	*ρ* = 1.1 × 10^−2^ Ω cm	66%	[30]
ITO	Ethanol	cw, 10.6 µm	PET	*R*_s_ ~ 400 Ω sq^−1^ (*ρ* = 12 × 10^−2^ Ω cm)	80%	[32]
ITO	Alcoholic solvent	ps, 1.75 µm	PET	*R*_s_ ~ 300 Ω sq^−1^	80%	[33]
ITO	Commercial suspension	cw, 1.5 µm	Glass	*R*_s_ ~ 80 Ω sq^−1^ (*ρ* = 1.3 × 10^−3^ Ω cm)	75%	[29]
ZnO	Ethanol, Ethylene glycol	ns, 248 nm	SiO_2_	*R* = 3.4 × 10^4^ MΩ → 78 MΩ	–	[42]
ZnO	1-pentanol	ps, 355 nm	Quartz	*R*_s_ ~ 5 kΩ sq^−1^ (*ρ* = 4.75 × 10^−2^ Ω cm)	84%	[44]
ZnO	Methanol, Butyl acetate	cw, 1065 nm	Glass, Na-CMC	*ρ* = 0.9 × 10^−4^ Ω cm	–	[55]
CuO	Ethylene glycol	cw & ns 1070 nm	Glass, PI	*ρ* = 31 × 10^−6^ Ω cm	–	[77]
CuO	IPA	ns, 1070 nm	Glass	*ρ* = 13 × 10^−6^ Ω cm	–	[78]
CuO	Ethylene glycol	ns, 1070 nm	PET	*ρ* = 30 × 10^−6^ Ω cm	–	[81]
CuO/NiO	Ethylene glycol	fs, 780 nm	Glass	–	–	[83]
CuO/GO	Water	cw, 405 nm	PET	*R*_s_ = 19.6 Ω sq^−1^	–	[84]
Cu_x_O	Isopropyl alcohol, Ethylene glycol	cw, 532 nm	Glass	*R* = over 10 000 Ω → 91 Ω	–	[85]
NiO_x_	Toluene	cw, 514.5 nm	Glass, PI	*R*_s_ = 655 Ω sq^−1^ (*ρ* = 63 × 10^−6^ Ω cm)	87%	[105]
NiO_x_	Toluene	cw, 532 nm	Glass	*ρ* = 103 × 10^−6^ Ω cm	–	[108]
NiO_x_	1-pentanol	cw, 532 nm	Glass, PI, PET	*R*_s_ = 53 Ω sq^−1^ (*ρ* = 98.8 × 10^−6^ Ω cm)	84%	[20]
NiOx	1-pentanol	cw, 532 nm	PET	–	–	[109]
NiO/Cr	Ethylene glycol	fs, 780 nm	Glass	*ρ* = 290 Ω cm		[110]

*ρ* resistivity, *R*_s_ sheet resistance, *R* resistance, *ns* nanosecond, *cw* continuous-wave, *ps* picosecond, *fs* femtosecond

The future LDP process of metal oxide nanomaterials is expected to expand both practical and theoretical aspects. The material used in the LDP process needs to be more diversified, and the application field needs to be further developed. Additionally, the reduction, sintering, and heat transfer phenomena that occur during the laser-induced modification of the physical, chemical properties of metal oxide NPs have to be theoretically explored more clearly. There are still several challenges and limitations related to the LDP process. However, the process obviously has unique advantages and is worth to be developed continuously as a method that can be used together with or as an alternative for conventional ones to produce next-generation transparent conductors, sensors, and electronics. Since the LDP process can be applied on the thermally vulnerable polymer substrates, we expect that the LDP process will become a mainstream tool for the development of flexible and stretchable electronics [111–120] in the future.

## Data Availability

The datasets used and/or analyzed during the current study are available from the corresponding author on reasonable request.

## Abbreviations

LDP: Laser digital patterning
NP: Nanoparticle
CW: Continuous-wave
CAD: Computer-aided design
ITO: Indium-doped din oxide
PET: Polyethylene terephthalate
IR: Infrared
UV: Ultraviolet
FET: Field-effect transistor
AZO: Al-doped ZnO
TEM: Transmission electron microscopy
*ρ*: Resistivity
*R*_s_: Sheet resistance
ns: Nanosecond
cw: Continuous-wave
ps: Picosecond
fs: Femtosecond
MPTS: 3-Methacryloxypropyl-trimethoxysilane
Na-CMC: Sodium carboxymethyl cellulose
APG: Atmospheric-pressure glow
IPL: Intense pulsed light
LRS: Laser-induced reductive sintering
PVP: Polyvinylpyrrolidone
EG: Ethylene glycol
PI: Polyimide
XRD: X-ray diffraction
T_g_: Glass transition temperature
GO: Graphene oxide
rGO: Reduced graphene oxide
NR: Nanorod
NW: Nanowire
CTAB: Cetyltrimethylammonium bromide
IPA: Isopanol
